# Extending BEHRT to UK Biobank: assessing transformer model performance in clinical prediction

**DOI:** 10.3389/fdgth.2026.1715506

**Published:** 2026-02-10

**Authors:** Yusuf Yildiz, Goran Nenadic, Meghna Jani, David A. Jenkins

**Affiliations:** 1 Faculty of Biology, Medicine and Health, School of Health Sciences, Division of Informatics, Imaging and Data Sciences, University of Manchester, Manchester, United Kingdom; 2 Department of Computer Science, University of Manchester, Manchester, United Kingdom; 3 Centre for Epidemiology Versus Arthritis, Centre for Musculoskeletal Research, University of Manchester, Manchester, United Kingdom; 4 NIHR Manchester Biomedical Research Centre, Manchester Academic Health Science Centre, Manchester University NHS Foundation Trust, Manchester, United Kingdom

**Keywords:** clinical prediction models, health informatics, large language models, medical terminology, modelling choices, transformers

## Abstract

**Introduction:**

Transformer-based models have shown strong potential for clinical prediction using electronic health record data, yet their performance can vary depending on modelling decisions and data characteristics.

**Methods:**

In this study, we trained a BEHRT model on hospital-based UK Biobank data and evaluated its performance across four clinical prediction tasks, including next-visit diagnosis and longer-term diagnosis prediction up to five years. We exhaustively assessed the impact of model size, medical terminology (CALIBER vs ICD-10), and data split strategies.

**Results:**

The large model consistently outperformed the smaller one in long-term prediction tasks (AUROC = 0.874 vs 0.858 at 5 years), while differences were marginal in 6-months prediction tasks. Performance was also sensitive to the vocabulary size, with CALIBER model yielding higher average precision scores (Average Precision Score = 0.773 vs 0.678 using ICD-10).

**Discussion:**

Our results show that transformer models can achieve high predictive performance across diverse clinical scenarios, but outcomes vary considerably depending on modelling choices, particularly in long-term prediction tasks.

## Introduction

There is a shift in healthcare from treating diseases after diagnosis to focusing on early detection, prevention, and intervention (1). To support this, Clinical Prediction Models (CPMs) are increasingly used in clinical decision-making (2). CPMs are statistical tools or algorithms that predict an individual's risk of currently having (diagnostic) or developing (prognostic) a medical condition, based on a defined set of predictors (3). These models have been developed for a range of clinical tasks, including predicting mortality risk (4), estimating cardiovascular disease risk (5), and or identifying individuals at risk of developing diabetes (6).

A growing source of data for CPMs is electronic health records (EHR) data, which document patients' medical histories across time (7, 8). With their scale, granularity, and longitudinal nature, EHR data offer significant potential to support clinical prediction. While early CPMs were predominantly based on statistical approaches like regression, the increasing richness and complexity of EHR data have encouraged the use of data-intensive machine learning models. Recent advancements in deep learning and large language modelling (LLM) have enabled the development of more sophisticated models that can capture the sequential and contextual nature of EHR data more effectively. For example, BEHRT (9)—Bidirectional Encoder Representations from Transformers for EHR—is a deep neural sequence transduction model based on the BERT (10) (Bidirectional Encoder Representations from Transformers) architecture, designed to generate contextualised embeddings from structured EHR data. BEHRT treats clinical codes as words, clinical visits as sentences, and a patient's full history as a document. It is pre-trained using a masked language modelling (MLM) objective, enabling it to learn contextual dependencies within a patient's timeline. The model architecture is flexible and accommodates multiple medical concepts, including diagnoses, medications, and measurements. In its original validation, BEHRT demonstrated promising predictive performance in predicting a patient's next diagnosis, including immediate, 6-month, and 12-month prediction windows, based on their longitudinal clinical history.

The domain of transformer-based clinical models has expanded rapidly from early BERT-based adaptations to a diverse ecosystem of generative and discriminative models. Foundational work such as Med-BERT (11) validated the importance of pre-training strategies on large-scale structured EHR data. More recently, the field has adapted more generative frameworks, such as Delphi-2M (12) and Foresight-2 (13) have used population-scale cohorts like the UK Biobank to forecast complex disease trajectories and synthesise longitudinal patient timelines. Concurrently, benchmarks of various pre-training objectives have revealed that optimal strategies are highly task-dependent (14). Addressing temporal robustness, the MOTOR study (15) introduced a time-to-event foundation model explicitly designed to mitigate the performance degradation caused by temporal distribution shifts. The relationship between model scale and clinical performance has also been explored by (16) and (17) who showed that scaling parameters improves prediction performance, but this depends on data quality and structure. Due to irregular presentation and evaluation of EHR foundational models, evaluation frameworks and reporting statements were developed (18–20).

Despite the successful application of BERT-based architecture in EHR modelling, several important considerations remain underexplored. For example, BEHRT was originally developed using CPRD (21), a large-scale primary care dataset in which diagnostic codes are classified in Read codes [“Read Codes,” (22)], later mapped to CALIBER (23, 24), a curated and standardised phenotyping framework tailored for epidemiological research. However, medical data is not always encoded using a single standard. Various medical terminologies such as Read, ICD (International Classification of Diseases [“International Classification of Diseases (ICD),” (25)], SNOMED-CT (26) and MedDRA (27) are used across different healthcare settings, with institutions adopting distinct conventions. Yet, the impact of this coding variation on clinical prediction performance remains unclear.

Our recent commentary (28) highlighted that data split strategies in LLM-based clinical prediction studies are often inconsistently applied, with limited investigation into their effects on model performance. In particular, the use of the full dataset for pre-training (e.g., MLM) followed by evaluation on overlapping data can introduce bias and weakens the robustness of real-world performance claims. This lack of standardisation in how data is allocated across training and validation phases prevents learning about how model works in unseen patients and presents a challenge for generalisability. Furthermore, large-scale transformer models are computationally demanding, which may limit their feasibility in resource-constrained healthcare environments. It remains unclear how models with reduced parameter sizes or simplified architectures perform in comparison, especially when considered for real-world deployment. We have therefore highlighted the need for a deeper understanding of these modelling decisions, particularly in terms of replicability across datasets, adaptability, and generalisability (28).

In this study, we train a BEHRT model using the UK Biobank and investigate how different model size, medical terminology, and data split strategies affect the predictive performance when the model is validated in next disease prediction tasks.

## Methods

### Data source

This study used the UK Biobank cohort. UK Biobank (29) is a large anonymised, linked dataset with half a million patient records including episode statistics providing structured longitudinal information on hospital visits, including demographic information, lab results, diagnostic codes.

Following the original BEHRT model built on CPRD, in this study we included all patients who had at least five documented hospital visit diagnoses. Diagnosis codes and patient age at the time of diagnosis were used as predictors, and no missing data were observed in the selected feature set. All diagnosis codes were encoded using ICD-10 standards, with some codes consisting of three alphanumeric characters and others extending up to five. To ensure consistency, all codes were dichotomised and standardised to a four-character format.

The number of patients included in each analysis varied depending on the prediction task and follow-up requirements. Detailed inclusion criteria for each task are described in the following section.

### BEHRT model architecture and input encoding

We reimplemented the BEHRT architecture and training procedure as originally proposed in (9). The aim was to establish a consistent baseline for comparison across different modelling decisions. Patient records were encoded as sequences of clinical visits, each containing diagnosis codes, patient age at the time of diagnosis, and visit year. Patients' diagnoses and corresponding ages were chronologically concatenated into a single sequence. A vocabulary list was created for the model to assign a unique index to each single diagnosis present in the dataset.

BEHRT follows a two-phase training approach. In the initial phase (pre-training), the model learns contextual representations of EHR sequences via the MLM objective. In the subsequent fine-tuning phase, the pre-trained model is adapted to specific prediction tasks (prediction of future diagnoses). Formally, for each patient p∈{1,2,3,4,………,P}, the EHR sequence is denoted as $V_{p}\;=\;\{v_{p}^{1},v_{p}^{2},v_{p}^{3},v_{p}^{4},v_{p}^{5},\ldots \ldots \ldots ,v_{p}^{n_{p}}\}$, where each visit $v_{p}^{j}$ contains diagnosis codes $\left\{d_{1},d_{2},d_{3},\ldots ,d_{m_{p}^{j}}\right\}$, drawn from the overall vocabulary $D=\;\{d_{i}{\}}_{i=1}^{G}$. To ensure uniform sequence length, padding (PAD) tokens were added. Visit boundaries were demarcated using [SEP] tokens, and the full patient sequence was preceded by a (CLS) token.

For prediction, the input sequence is defined as:

$$x_{p}\;=\;\{V_{p}^{1},V_{p}^{2},\ldots ,\;V_{p}^{j}\},y_{p}=\;w_{\;j+1}$$

where $x_{p}$ comprises visits up to *j*, and $y_{p}$ is a multi-hot vector representing diagnoses in the (j+1)th visit. The model's output $y_{p}^{*}$ represents the predicted probabilities for each disease.

**Figure 1:**
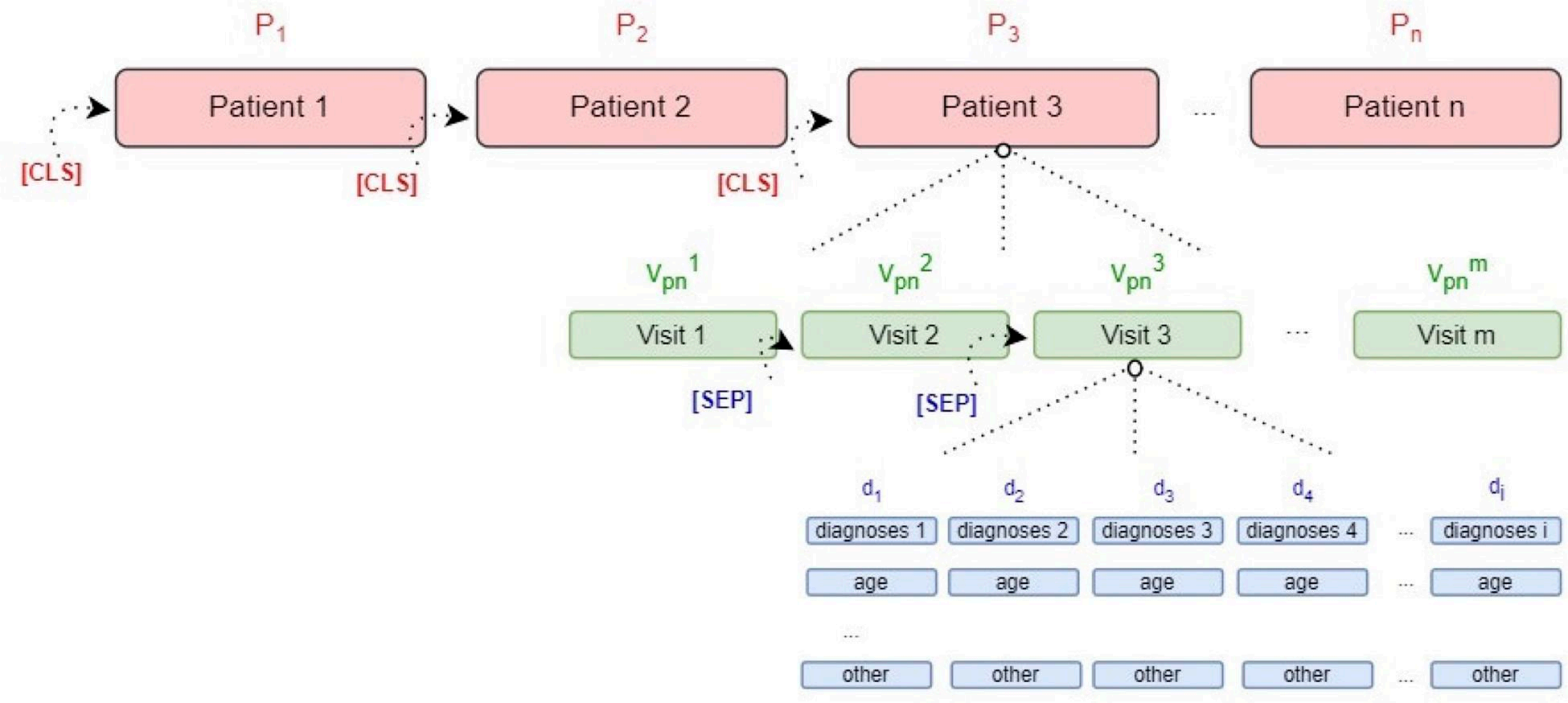
Schematic representation of the BEHRT input structure using patient EHR data. Each patient sequence (P) begins with a classification token (CLS) and consists of a chronological series of visits (Vₚⁿ). Each visit is separated by a visit-level separator token (SEP) and contains structured medical information, including diagnosis codes (d_1_ to d_i_), the patient's age at the time of diagnosis, and other visit-level attributes.

**Figure 2:**
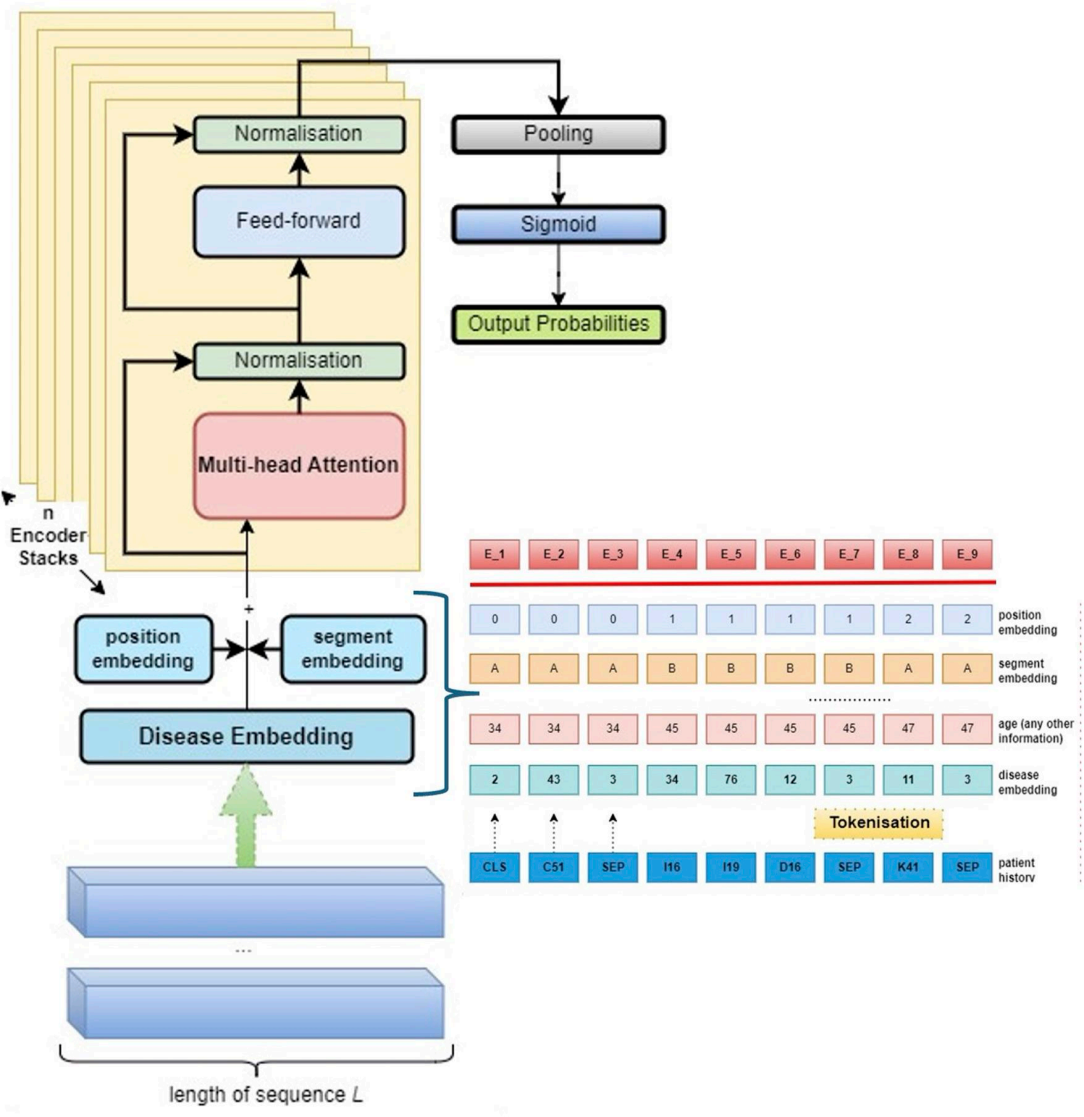
Overview of the full end-to-end BEHRT model architecture and patient embedding pipeline. The input consists of a patient's medical history, tokenised into sequences of diagnosis codes along with auxiliary features such as age. Each token is enriched with position and segment embeddings, then passed through multiple stacked Transformer encoder layers. The contextualised embeddings are pooled and processed through a sigmoid layer to produce output probabilities for the prediction tasks. Adapted with permission from “BEHRT architecture” by Yikuan Li, Shishir Rao, José Roberto Ayala Solares, Abdelaali Hassaine, Rema Ramakrishnan, Dexter Canoy, Yajie Zhu, Kazem Rahimi and Gholamreza Salimi-Khorshidi, licensed under CC BY 4.0.

A schematic of the input structure is provided in Figure 1, and the full BEHRT architecture used is visualised in Figure 2. To further illustrate input process, a step-by-step example of a representative patient sequence, transforming raw visit data into tokenised encodings, is provided in Supplementary Tables S6, S7. For further technical details, readers are referred to (9).

### Prediction task selection

We evaluated model performance across four prediction tasks:

Task 1: predicting diagnoses at the patient's next clinical visit**;**
Task 2: predicting diagnoses occurring within 6-months;
Task 3: predicting diagnoses within the next 12-months;
Task 4: predicting diagnoses within a 60-months (5-year).

The first three tasks were considered by Li et al.(2019), while the fourth task was introduced to include a longer-term prediction window motivated by two key considerations: (i) many clinical models aim to predict long-term outcomes, and (ii) datasets derived from hospital admission records often contain less frequent and more episodic documentation of patient visits compared to those derived from primary care, which tend to capture more regular and continuous healthcare interactions.

The number of patients varied across modelling tasks due to differences in task-specific inclusion criteria. During the pre-training phase, as the model does not require any label, all patients that have at least five visits in their hospital records were included. However, the prediction tasks (i.e., fine-tuning phase) required stricter eligibility. In these tasks, a patient must have at least one future hospital visit after the defined prediction point, and that visit must fall within the target prediction window. Therefore, an observable diagnosis during follow-up was necessary to compute the evaluation metrics. As a result, patients without sufficient follow-up outcome events (6-months, 12-months) were excluded, yielding progressively smaller cohorts for each prediction scenario.

### Experimental setup

We trained BEHRT on UK Biobank data to establish a baseline using CALIBER terminology, the original architecture, and the data split strategy described by (9). This allowed us to assess the generalisability of the model to a new dataset. To evaluate the impact of key modelling decisions, we then trained and compared variants of BEHRT under three conditions:

Model size: small and large configurations, with hyperparameters summarised in Table 1.
Medical terminology: CALIBER (43 phenotypes) vs. ICD-10 (9,217 codes).
Data split strategy: Split 1, where the full dataset was used for pre-training followed by an 80/20 train-test split for fine-tuning, and Split 2, where 50% of the dataset was used for pre-training and the remaining 50% was split 80/20 for fine-tuning.

**Table 1:** Parameter configurations used for the small and large BEHRT models.

Parameters	Small size model	Large size model
Hidden size	150	288
Dropout for hidden layer	0.3	0.1
Multi-head attention layers	4	6
Attention heads	6	12
Dropout for attention	0.4	0.1
Intermediate layer size	108	512

### Evaluation metrics

To assess model performance, we used two evaluation metrics: Area Under the Receiver Operating Characteristic Curve (AUROC) (30) and Average Precision Score (APS) (31). AUROC captures the model's ability to discriminate between positive and negative cases, while APS reflects a weighted average of precision and recall across different thresholds. Both metrics were calculated on a per patient basis and averaged across all patients to provide a robust overall performance measure.

### Disease level analysis

To assess disease-level performance, we analysed the top 20 most prevalent diseases for each prediction task. In order to handle the high dimensionality of the output space, we utilised a Threshold Optimization strategy based on the Precision-Recall curve. Because standard decision thresholds (*τ* = 0.5) would be too high for such output space, we identified the optimal probability threshold that maximized the F1-score and reported the Adjusted Recall and Adjusted Precision at this calibrated point for each disease. For this analysis we have used the following experimental setup: Large Model/Split 1/ICD-10.

## Results

### Study population

The study population was derived from hospital episode records linked to the UK Biobank cohort. Patients were eligible for inclusion if they had at least five recorded hospital visits. The final pretraining cohort consisted of 203,638 patients under the ICD-10 and 189,803 under CALIBER phenotyping. The discrepancy between CALIBER and ICD-10 cohorts is primarily due to the mapping process, where a subset of ICD-10 codes could not be mapped to CALIBER phenotypes and were therefore excluded from CALIBER-based experiments.

Table 2 summarises the number of patients included for each of the four prediction tasks across both coding systems. The reduction in patient numbers across tasks is attributable to follow-up requirements and target label availability.

**Table 2:** Number of patients included in pretraining and prediction tasks using CALIBER and ICD-10 terminologies. Pretraining includes all eligible patients with at least five hospital visits.

Development stage	CALIBER	ICD-10
Pretraining	189,803	203,638
Task 1	189,803	203,638
Task 2	173,604	186,505
Task 3	164,799	177,422
Task 4	110,480	120,779

### Experiments

It is important to clarify that any results related to the model trained using CPRD, were taken directly from the (9), and no additional experiments were performed on CPRD in this work. Subsequent analyses were conducted only using the UK Biobank dataset.

### MLM results

During pre-training, models were trained exclusively on UK Biobank data using the masked language modelling objective. Across these experiments, the large model consistently achieved higher APS scores than the small model, indicating that larger model capacity supports more effective representation learning. The highest performance (APS = 0.497) was observed with CALIBER terminology under Split 1, whereas the lowest (APS = 0.322) was found under the ICD-10 setting.

For context (9), reported a higher APS of 0.659 in their CPRD-based BEHRT experiments, reflecting the influence of larger datasets and broader vocabulary coverage. While not directly comparable, this benchmark provides useful perspective on the effect of dataset characteristics.

### Model size effect: large vs. small model configurations

For Tasks 1 to 3 (covering prediction windows up to 12 months), performance differences between the two configurations were minimal, with the large model outperforming the small model by less than 1% in AUROC and APS across all settings (see Table 3). However, the increased model capacity became more evident in Task 4 (5-year prediction). In particular, under the CALIBER and Split 2 setting, the large model achieved an AUROC of 86.7% compared to 63.4% for the small model, suggesting that larger models may be better suited for capturing long-term dependencies.

**Table 3:** Predictive performance of BEHRT models across experimental configurations. Note that APS and AUROC values reported here are patient-level averages: metrics are calculated for each patient individually and then averaged across the entire validation cohort.

Terminology	Data Split	Model Size	MLM	Task 1		Task 2		Task 3		Task 4	
			APS	AUROC	APS	AUROC	APS	AUROC	APS	AUROC	APS
CALIBER	SP1	Large	0.497	0.841	0.375	0.948	0.773	0.929	0.688	0.874	0.659
CALIBER	SP1	Small	0.432	0.835	0.381	0.943	0.769	0.923	0.687	0.858	0.634
CALIBER	SP2	Large	0.485	0.837	0.373	0.945	0.766	0.924	0.678	0.867	0.642
CALIBER	SP2	Small	0.451	0.828	0.371	0.944	0.784	0.923	0.701	0.634	0.634
ICD-10	SP1	Large	0.353	0.953	0.045	0.984	0.678	0.979	0.415	0.951	0.311
ICD-10	SP1	Small	0.322	0.952	0.045	0.977	0.678	0.971	0.415	0.911	0.299

Notably, the largest AUROC value across all experiments was recorded by the large model under the ICD-10 setting (AUROC: 0.953). However, this coincided with the lowest APS score (0.045), highlighting the difficulty of achieving precise predictions when using ICD-10, which in our study included 9,217 unique codes, compared with only 43 codes under CALIBER.

### Effect of data split strategy on model performance

As expected, Split 1 resulted in slightly higher performance across most tasks. This difference was most evident in Task 4, where the AUROC dropped from 85.8% with Split 1 to 63.4% under Split 2. These findings suggest that data splits play a significant role in performance outcomes, and that reusing the same data for both pre-training and fine-tuning may inflate results due to potential data leakage.

### Medical terminology effect: CALIBER vs. ICD-10 vocabulary

Models trained with CALIBER (43 vocabulary) consistently achieved higher APS scores, whereas those trained with ICD-10 (9,217 vocabulary) showed higher AUROC values under the same setup (UK Biobank, Split 1). For instance, in Task 4, the CALIBER model (large) achieved an AUROC of 87.4% and an APS of 65.9%, whereas the ICD-10 model reached 95.1% AUROC but only 31.1% APS. This pattern was consistent across all tasks and both model sizes. Notably, in Task 1, both ICD-10 models produced an APS of only 0.045, highlighting the difficulty of making accurate predictions under sparse and fine-grained terminology systems. These findings shows that while ICD-10-based models retain strong discrimination capability (as reflected by high AUROC), they are less precise in identifying relevant outcomes, especially in tasks with limited label density. The observed trade-off reflects the influence of label sparsity and class imbalance introduced by large terminologies.

### Effect of task selection

This analysis aimed to evaluate how model performance varies across different prediction windows, offering insight into clinically meaningful task design. Across all settings and configurations, the model consistently achieved the highest predictive performance in Task 2 (6-month prediction), followed by Task 3 (12-month), Task 4 (60-month), and lastly Task 1 (next-visit prediction). This trend was observed in both AUROC and APS metrics. For example, under the CALIBER vocabulary, using the large model and split 2, AUROC scores for Tasks 1–4 were 83.7%, 94.5%, 92.4%, and 86.7%, respectively. Corresponding APS scores were 37.3%, 76.6%, 67.8%, and 64.2%.

### Disease level performance

Descriptive statistics for the top 20 diseases in the pre-training cohort are provided in Supplementary Table S1. Task specific model's performance in disease-level and frequency of top 20 diseases within validation set is presented in Supplementary Tables S2–S5. Note that for these disease-level metrics, we report the performance of the Large Model trained on Data Split 1 using the ICD-10 terminology. This configuration was selected to demonstrate performance at the most granular vocabulary level, avoiding the aggregation inherent in mapped phenotypes. Disease-level analysis (Supplementary Tables S2–S5) reveals that model performance is dependent on the prediction window and disease phenotype. Across all tasks, optimal decision thresholds were consistently low (0.005 < *τ* < 0.09), confirming that the model's confidence is low due to both sparsity and high outcome options. However, once calibrated, the model demonstrated good level sensitivity.

We observed that predictive performance generally improved with the length of the observation window. For example, for Essential Hypertension (I10), Adjusted Recall rose from 0.06 in the 6-month task (Task 2) to 0.948 in the 5-Year task (Task 4). A similar pattern was observed for Unspecified Arthrosis condition (M199) where Adjusted Recall increased from 0.05 in the 6-month task (Task 2) to 0.62 in the 5-Year task (Task 4). This may suggest the model aggregates long-term risk profiles despite the noise in individual acute visits. On the other hand, high prediction performance was observed for “History” codes (e.g., Z864, chapter Z for ICD-10 codes) that shows memory retention capability of the models.

## Discussion

This study demonstrates how modelling decisions—such as the choice of clinical coding, model size and data split—influence the predictive performance of transformer-based models trained on real-world hospital episode statistics data. By re-training BEHRT on UK Biobank and varying the parameters, we found that these choices can affect both accuracy and precision, especially in long-term prediction tasks. Notably, models using CALIBER terminology achieved markedly higher APS scores than those using ICD-10, while smaller models performed nearly as well as larger ones for short-term predictions. Additionally, some studies in the literature supports our findings and indicate that architectural modifications and different modelling decisions such as hyperparameter tuning, training data selection, and the integration of diverse data types significantly impact accuracy (32–34).

It is important to consider the inherent differences between the datasets used in this line of research. For instance, CPRD is substantially larger (∼7 million patients) than UK Biobank (∼500,000 patients) and contains more frequent primary care records, whereas UK Biobank, based on voluntary participation, represents a narrower and older population, with limited coverage of younger age groups and related conditions (35). Such differences influence the size of the disease vocabulary (e.g., 301 classes in CPRD vs. 43 in UK Biobank) and, in turn, affect commonly used metrics. Smaller vocabularies generally yield higher APS because predictions are made over fewer negative classes (36), while AUROC, though less sensitive to class imbalance, is influenced by dataset complexity. Results reported in (9) for CPRD showed higher AUROC compared to our UK Biobank experiments, reflecting these differences. This underlines that standard metrics can be misleading across datasets with distinct structures, reinforcing the need for evaluation measures that better account for real-world EHR sparsity (37).

### Model size effect

Increasing model size led to moderate improvements in prediction performance, particularly for longer-term clinical prediction tasks. In Task 4, the AUROC score improved from 63.4% to 86.7%. This trend is consistent with broader machine learning literature, where larger models demonstrate a greater capacity to capture complex, non-linear relationships over extended input sequences (38, 39). In the context of clinical prediction, longer forecast horizons likely require capturing more subtle, temporally distant dependencies within the data, for which increased model complexity is beneficial. However, for shorter prediction windows, such as in Task 2, the performance gains were marginal. The AUROC scores for the large and small models were 94.8% and 94.3%, respectively, and APS scores were 77.3% and 76.9%, indicating a modest benefit.

However, increasing model size does not universally guarantee improved performance. In settings where the dataset is limited in size or diversity, larger models may overfit and fail to generalise (11). Our findings suggest that for near-term clinical prediction tasks (e.g., within 6–12 months), smaller models may offer comparable performance to larger models, with much lower computational demands. This could make them a practical choice for deployment in resource-constrained environments or embedded clinical systems. This finding on model size aligns with recent scaling analyses by (40) confirming that for discriminative tasks on sparse structured data, optimizing input representation often yields greater gains than simply increasing parameter scale. Furthermore, regarding sequence length (41), evaluated the utility of long-context models, finding that longer context models improve predictive performance, but very long sequences and multimodal data simultaneously still pose scalability challenges.

### Medical terminology effect

**Figure 3:**
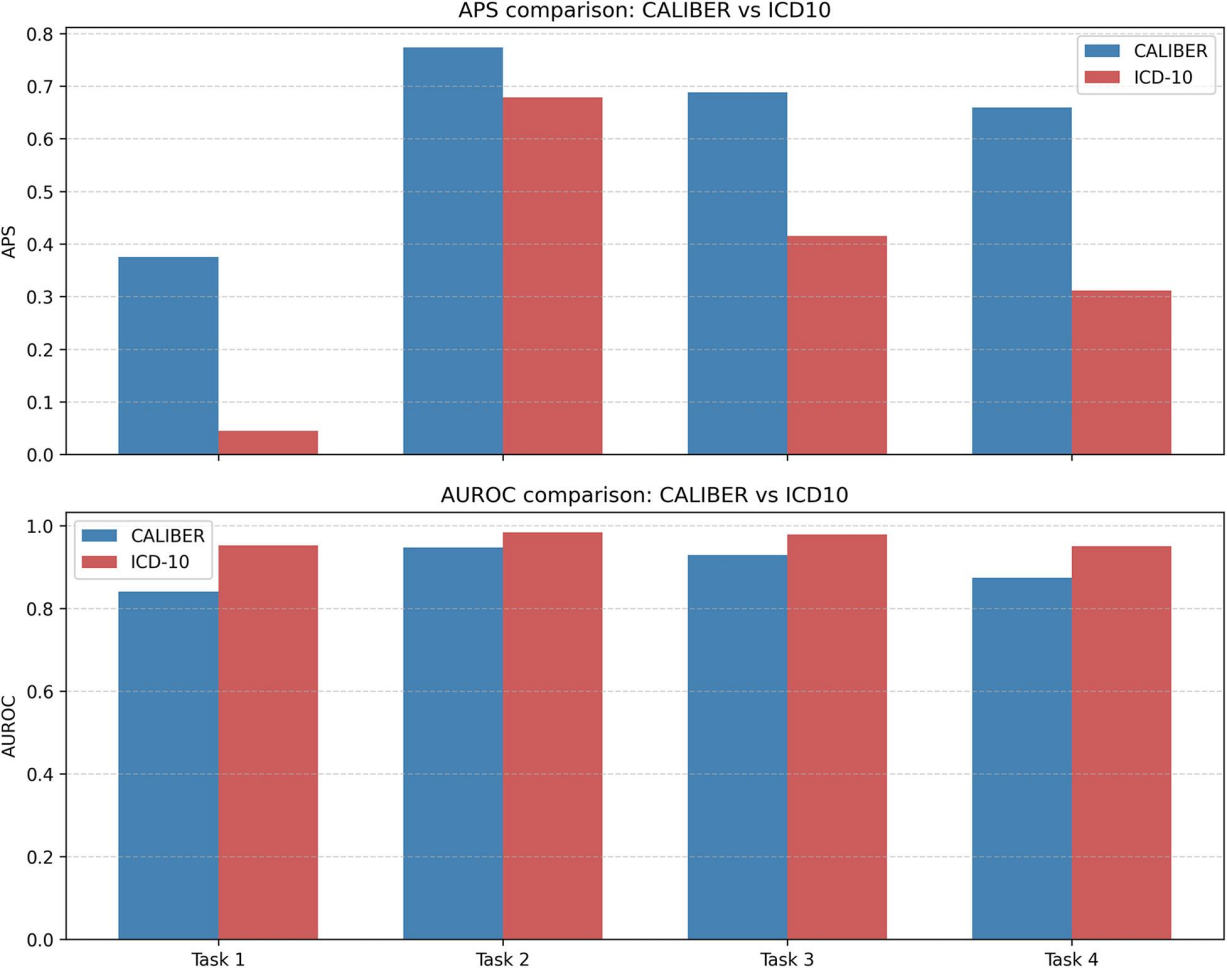
Comparison of Model Performance Using CALIBER and ICD-10 Terminologies Across Prediction Tasks. Bar plots show average precision score (APS, top) and area under the receiver operating characteristic curve (AUROC, bottom) for each task using CALIBER (blue) and ICD-10 (red) vocabularies. Results are based on the large model configuration using data split 1. APS reflects the precision of predictions at different recall thresholds, while AUROC represents the model's overall discriminative ability.

Another important factor influencing model performance was the choice of clinical terminology system. As shown in Figure 3, models trained on smaller, curated vocabularies such as CALIBER consistently achieved higher APS scores. This reflects the advantage of reduced class sparsity and increased disease prevalence per class. In contrast, using a larger terminology like ICD-10 introduced significant sparsity. Although this resulted in higher AUROC scores, APS values were notably lower. AUROC may appear artificially optimistic in sparse outcome distributions, whereas APS is more sensitive to the model's ability to make accurate positive predictions (36, 42). Therefore, careful consideration of vocabulary size and structure is essential when developing and evaluating clinical prediction models. Vocabulary design must align with clinical needs. Excessively reducing the number of codes may lead to oversimplification, while overly detailed terminologies may introduce noise without meaningful benefit.

On the other side, literature [e.g., (43–45)] shows that incorporating domain knowledge through medical ontologies and leveraging the hierarchical structure of clinical terminologies may offer a way to retain rich vocabularies without compromising performance. Such approaches could enable better balance between model granularity and prediction accuracy.

### Data split strategy effect

Different strategies for splitting data between pre-training and fine-tuning phases have been explored to optimise model generalisability and avoid overfitting. In this study, we compared two approaches: using the entire dataset for pre-training followed by an 80/20 split for fine-tuning (Split 1), and an alternative where only 50% of the data was used for pre-training and the remaining 50% split for fine-tuning (Split 2). Although overall performance differences between these strategies were modest in the large models, a notable drop in AUROC was observed in the small model for Task 4 when applying Split 2, with a decrease from 85.8% to 63.4%.

These findings align with the expectation that maximising the amount of data used during pre-training improves the richness of the learnt representations (10). From a theoretical standpoint, these findings are consistent with neural scaling laws, which demonstrate that the performance of deep learning models improves predictably with increasing model size, training dataset size, and computational resources (38). Such laws often follow power-law behaviours, where performance gains scale smoothly with larger pre-training corpora and more parameters (38).

In practice, transformer-based models like BEHRT benefit substantially from increased pre-training data, as the bulk of representation learning occurs during this phase. Even when fine-tuning data is limited, the representations acquired through broad pre-training enable effective downstream prediction. However, it remains crucial to ensure that fine-tuning datasets are properly separated from the pre-training data to avoid overfitting and inflation of the performance.

### Task selection

Selecting appropriate prediction windows is crucial for clinical relevance and model performance. In our experiments, shorter-term predictions, particularly the 6- and 12-month horizons, consistently achieved higher AUROC and APS scores, while next-visit prediction proved the most challenging where labels are sparse and only have one positive value. This likely reflects the inherent sparsity and unpredictability of next-visit events, where clinical labels are fewer and more variable, reducing model learnability.

Conversely, longer windows, such as 6 or 12 months, allow more chronic disease progression to manifest, increasing the density of positive outcomes and improving predictive signal. However, extending the window further to 60 months introduced greater uncertainty, balancing the benefits of label density with the complexity of long-term clinical trajectories. These findings highlight the importance of aligning prediction windows with clinical objectives, ensuring a balance between early risk identification and achievable prediction accuracy. Table 4 provides summary of the insight about the selected task's reliability and the reasons behind it.

**Table 4:** Summary of prediction task difficulty and underlying characteristics. Relative difficulty of each prediction window based on observed model performance. Tasks are ranked from hardest to easiest, considering clinical data sparsity, label density, and predictability of outcomes.

Task	Difficulty	Reason
Next Visit	Least reliable (lowest AUROC/APS)	Sparse/noisy, few labels, unpredictable
60 Months	More reliable than next visit, but less than 6–12 months	Long horizon introduces uncertainty
12 Months	Reliable	Diseases develop within a manageable, predictable timeframe
6 Months	Most reliable (highest AUROC/APS)	Good label density, clinically relevant window, reduced noise

### Disease level analysis

Our granular analysis showed a confidence gap driven by the high dimensionality of the prediction matrix. We found out that the model is prone to “probability dilution,” where the probability mass is distributed across many plausible codes, preventing correct predictions from crossing standard decision thresholds. Therefore, our results highlight a divergence between global ranking metrics (AUROC ∼0.95) and uncalibrated decision metrics. The high AUROC indicates the model successfully ranks the correct diagnosis higher than noise. However, the low absolute probabilities observed in our granular analysis reflect the “sparsity bias” of training on episodic hospital data. Clinically, this implies that transformer models trained on HES data function best as Risk Ranking Engines rather than definitive binary classifiers, unless disease-specific threshold calibration is applied.

### Limitations

While this study provides important methodological insights, several limitations must be acknowledged. First, although we compare results from models trained on UK Biobank with previously reported results from BEHRT trained on CPRD, we did not conduct new experiments on CPRD. This means that any cross-dataset observations are indirect, relying on published results rather than side-by-side experiments on both datasets. Consequently, these comparisons should be interpreted cautiously.

Second, due to the inclusion criteria of the study, patients with incomplete follow-up or fewer than five visits were excluded. This introduces survivor bias, that is patients who remain in the dataset longer and generate more records are more likely to be included and modelled. While such bias is common in real-world EHR studies, this work is intended as a methodological contribution rather than for immediate clinical deployment. As discussed in our previous work (28), further steps are necessary to evaluate bias and ensure fairness in downstream implementation.

Finally, the study explored only a focused set of modelling configurations due to computational and time resource constraints. Although these experimental choices were guided by practical relevance and alignment with prior literature, we did not experimentally evaluate every possible permutation of the comparisons (e.g., pairing every terminology size with every data split strategy). Instead, the experiments were selected to sufficiently demonstrate the core concepts regarding splitting strategies and vocabulary size. Therefore, the findings should not be considered exhaustive. A more comprehensive, simulation-based exploration of modelling strategies may be pursued in future work, although such studies remain constrained by the computational demands of training large transformer-based architectures.

### Future direction

Several avenues for future research emerge from this work. First, alternative pre-training or fine-tuning strategies, such as survival-based objectives (15) could be explored to better capture longitudinal dependencies in EHR data. Given the near-comparable performance of smaller models, further investigation into lightweight architectures would be valuable, particularly for deployment in resource-constrained clinical environments. In addition, technical time adjustment such as dynamic prediction windows, rather than static time frames, may better reflect the irregular nature of real-world clinical follow-up and could improve predictive robustness. Important future research could be related to how the hierarchical nature of clinical terminologies is utilised within these models. Current approaches often involve dichotomising detailed codes, such as collapsing seven-character ICD codes into broader four-character groupings. However, this may result in a loss of clinically relevant granularity and obscure the rich taxonomical relationships embedded in the terminologies. Future work could systematically investigate whether more sophisticated representations that preserve or exploit the full hierarchical structure (e.g., multi-level encoding strategies for ICD, Read, or SNOMED codes) could increase model performance, and interpretability (43, 44, 46, 47).

While this study focuses on the BEHRT architecture we acknowledge the rapid advancement of generative LLMs in clinical reasoning tasks (48–50); however our findings is related to the specific utility of discriminative encoders for high-dimensional structured prediction. Decoder-based LLMs presents distinct challenges and require extensive serialization of patient history while BEHRT processes structured clinical codes directly as tokens, preserving the precise taxonomy of systems like ICD-10. Furthermore, while generative models excel at reasoning, they are prone to “hallucinating” (51, 52) unless strictly fine-tuned. Future research should investigate hybrid approaches that combine the representation learning strengths of encoder models with the reasoning capabilities of generative LLMs.

## Conclusion

This study examined how key modelling decisions influence the predictive performance of a transformer-based model trained on UK Biobank data. Model behaviour was sensitive to variations in model size, terminology, and data split strategy, each affecting predictive accuracy across tasks. CALIBER, a curated terminology, yielded higher precision scores, while ICD-10, with its larger and sparser vocabulary, produced higher AUROC but substantially lower APS. Larger models improved long-range predictions but offered only marginal gains for short-term tasks.

This work provides methodological insights into how transformer-based models can be adapted for diverse healthcare datasets and highlights the trade-offs involved in key modelling decisions. By clarifying the impact of terminology, model complexity, and data partitioning strategies, our findings strengthen the evidence base for building prediction models that are not only technically robust but also clinically meaningful. Ultimately, these insights help pave the way toward more trustworthy and applicable AI systems that can better support decision-making in real-world healthcare.

## Data Availability

The core transformer architecture used in this study is based on the publicly available BEHRT implementation, accessible at https://github.com/deepmedicine/BEHRT. Data processing pipelines, experimental configurations, and analysis scripts developed for this study are available at https://github.com/yildizyy/BEHRT-UKBiobank. Please note that due to data privacy restrictions, specific data extraction scripts tied to the secure research environment are not included; however, the processing logic is fully explained in methods section to facilitate replication on independent datasets. Requests to access these datasets should be directed to https://www.ukbiobank.ac.uk.
