# Supplementary material for: Extending BEHRT to UK Biobank: assessing transformer model performance in clinical prediction
Source: Front Digit Health. 2026 Feb 10;8:1715506. doi: 10.3389/fdgth.2026.1715506 (PMC12929515; doi:10.3389/fdgth.2026.1715506)
Supplement: Supplementary file 1 [file Datasheet1.pdf]

Supplementary Table S1. Frequency and prevalence of the top 20 most represented diseases within the Masked Language Modelling (MLM) pre-training cohort.

**Rank**	**Disease ICD-10 Code**	**Disease Description**	**Patient Count (N)**	**Prevalence (%)**	**Total Mentions**
**1**	I10	Essential (primary) hypertension	103086	50.62	103086
**2**	Z864	Personal history of psychoactive substance abuse	56426	27.71	56426
**3**	E780	Pure hypercholesterolaemia	53762	26.40	53762
**4**	K573	Diverticular disease of large intestine without perforation or abscess	41452	20.36	41452
**5**	K449	Diaphragmatic hernia without obstruction or gangrene	40566	19.92	40566
**6**	Z922	Personal history of long-term (current) use of other medicaments	36015	17.69	36015
**7**	M199	Arthrosis, unspecified	35632	17.50	43143
**8**	Z867	Personal history of diseases of the circulatory system	34340	16.86	34340
**9**	K219	Gastro-oesophageal reflux disease without oesophagitis	32556	15.99	32556
**10**	Z871	Personal history of diseases of the digestive system	32545	15.98	32545
**11**	J459	Asthma, unspecified	32127	15.78	32127
**12**	E119	Type 2 diabetes mellitus without complications	30465	14.96	30465
**13**	R074	Chest pain, unspecified	29818	14.64	29818
**14**	Z921	Personal history of long-term (current) use of anticoagulants	29077	14.28	29077
**15**	I251	Atherosclerotic heart disease	28155	13.83	28155
**16**	E669	Obesity, unspecified	26558	13.04	26558
**17**	M179	Gonarthrosis, unspecified	25400	12.47	25400
**18**	K590	Constipation	25108	12.33	25108
**19**	I259	Chronic ischaemic heart disease, unspecified	25073	12.31	25073
**20**	M139	Arthritis, unspecified	24652	12.11	28575

Supplementary Table S2. Disease-level performance metrics (APS, Adjusted Precision, and Adjusted Recall) and optimal thresholds for the top 20 most prevalent diseases in the Next Visit Prediction task (Task 1). Results correspond to the Large Model configuration using Data Split 1 and ICD-10 terminology.

Rank	N	Code	Disease Name	APS	Adj. Precision	Adj. Recall	Opt. Thresh
**1**	576	K573	Diverticular disease of large intestine without perforation or abscess	0.012	0.014	0.976	0.0129
**2**	548	I10	Essential (primary) hypertension	0.014	0.015	0.162	0.0142
**3**	425	Z864	Personal history of psychoactive substance abuse	0.01	0.01	0.995	0.0107
**4**	386	M199	Arthrosis, unspecified	0.009	0.01	0.702	0.0091
**5**	366	H269	Cataract, unspecified	0.007	0.009	0.989	0.0087
**6**	347	E780	Pure hypercholesterolaemia	0.008	0.009	0.997	0.0081
**7**	342	K449	Diaphragmatic hernia without obstruction or gangrene	0.007	0.008	0.994	0.0078
**8**	328	R69	Unknown and unspecified causes of morbidity	0.01	0.011	0.323	0.0077
**9**	314	Z871	Personal history of diseases of the digestive system	0.007	0.008	0.997	0.0073
**10**	289	K219	Gastro-oesophageal reflux disease without oesophagitis	0.006	0.007	0.995	0.0072
**11**	280	Z961	Presence of intraocular lens	0.006	0.007	0.995	0.0071
**12**	272	K635	Polyp of colon	0.006	0.007	0.923	0.0065
**13**	265	Z922	Personal history of long-term (current) use of other medicaments	0.007	0.009	0.034	0.0065
**14**	256	Z867	Personal history of diseases of the circulatory system	0.008	0.021	0.023	0.0062
**15**	254	M179	Gonarthrosis, unspecified	0.005	0.006	0.995	0.0063
**16**	251	M139	Arthritis, unspecified	0.005	0.006	0.995	0.0059
**17**	244	Z966	Presence of orthopaedic joint implants	0.006	0.006	0.586	0.0059
**18**	240	K297	Gastritis, unspecified	0.005	0.006	0.988	0.0056
**19**	225	E669	Obesity, unspecified	0.005	0.007	0.049	0.0055
**20**	223	Z538	Procedure not carried out for other reasons	0.006	0.01	0.018	0.0051

Supplementary Table S3. Disease-level performance metrics (APS, Adjusted Precision, and Adjusted Recall) and optimal thresholds for the top 20 most prevalent diseases in the 6-Month Prediction task (Task 2). Results correspond to the Large Model configuration using Data Split 1 and ICD-10 terminology.

Rank	N	Code	Disease Name	APS	Adj. Precision	Adj. Recall	Opt. Thresh
**1**	563	Z864	Personal history of psychoactive substance abuse	0.019	0.029	0.08	0.0147
**2**	546	I10	Essential (primary) hypertension	0.019	0.031	0.053	0.0146
**3**	457	Z871	Personal history of diseases of the digestive system	0.016	0.047	0.037	0.012
**4**	442	Z511	Chemotherapy session for neoplasm	0.012	0.013	0.387	0.0107
**5**	440	E780	Pure hypercholesterolaemia	0.013	0.015	0.1	0.0115
**6**	403	Z867	Personal history of diseases of the circulatory system	0.018	0.033	0.084	0.0114
**7**	394	Z922	Personal history of long-term (current) use of other medicaments	0.012	0.015	0.096	0.01
**8**	375	Z921	Personal history of long-term (current) use of anticoagulants	0.014	0.03	0.043	0.0092
**9**	374	N390	Urinary tract infection, site not specified	0.016	0.038	0.045	0.0094
**10**	355	K573	Diverticular disease of large intestine without perforation or abscess	0.009	0.01	0.651	0.0096
**11**	352	D649	Anaemia, unspecified	0.015	0.024	0.091	0.0094
**12**	332	K590	Constipation	0.016	0.027	0.127	0.0093
**13**	332	M199	Arthrosis, unspecified	0.009	0.014	0.045	0.0094
**14**	318	I251	Atherosclerotic heart disease	0.007	0.009	0.995	0.008
**15**	317	Z501	Other physical therapy	0.016	0.049	0.095	0.0073
**16**	300	R11	Nausea and vomiting	0.01	0.014	0.117	0.008
**17**	295	Z961	Presence of intraocular lens	0.007	0.008	0.997	0.0077
**18**	293	K449	Diaphragmatic hernia without obstruction or gangrene	0.007	0.009	0.13	0.0077
**19**	285	K219	Gastro-oesophageal reflux disease without oesophagitis	0.009	0.011	0.102	0.0076
**20**	267	N179	Acute renal failure, unspecified	0.018	0.032	0.131	0.0068

Supplementary Table S4. Disease-level performance metrics (APS, Adjusted Precision, and Adjusted Recall) and optimal thresholds for the top 20 most prevalent diseases in the 12-Month Prediction task (Task 3). Results correspond to the Large Model configuration using Data Split 1 and ICD-10 terminology.

Rank	N	Code	Disease Name	APS	Adj. Precision	Adj. Recall	Opt. Thresh
**1**	844	I10	Essential (primary) hypertension	0.025	0.028	0.211	0.023
**2**	811	Z864	Personal history of psychoactive substance abuse	0.023	0.023	0.995	0.022
**3**	653	Z871	Personal history of diseases of the digestive system	0.018	0.019	0.985	0.0174
**4**	637	E780	Pure hypercholesterolaemia	0.02	0.021	0.419	0.0185
**5**	599	K573	Diverticular disease of large intestine without perforation or abscess	0.019	0.022	0.135	0.0154
**6**	598	Z867	Personal history of diseases of the circulatory system	0.027	0.038	0.154	0.0174
**7**	588	M199	Arthrosis, unspecified	0.023	0.031	0.129	0.0163
**8**	539	Z922	Personal history of long-term (current) use of other medicaments	0.024	0.045	0.067	0.0154
**9**	519	Z921	Personal history of long-term (current) use of anticoagulants	0.03	0.042	0.154	0.0144
**10**	481	K219	Gastro-oesophageal reflux disease without oesophagitis	0.016	0.027	0.089	0.0128
**11**	473	K449	Diaphragmatic hernia without obstruction or gangrene	0.015	0.017	0.131	0.0127
**12**	450	M139	Arthritis, unspecified	0.014	0.015	0.262	0.0114
**13**	437	Z511	Chemotherapy session for neoplasm	0.012	0.013	0.57	0.0112
**14**	417	K590	Constipation	0.019	0.036	0.098	0.0111
**15**	415	D649	Anaemia, unspecified	0.016	0.026	0.116	0.0122
**16**	402	R11	Nausea and vomiting	0.016	0.021	0.139	0.0101
**17**	398	N390	Urinary tract infection, site not specified	0.022	0.044	0.075	0.0126
**18**	398	R074	Chest pain, unspecified	0.012	0.012	0.558	0.01
**19**	386	Z538	Procedure not carried out for other reasons	0.014	0.018	0.171	0.0107
**20**	385	Z961	Presence of intraocular lens	0.011	0.013	0.223	0.0111

Supplementary Table S5. Disease-level performance metrics (APS, Adjusted Precision, and Adjusted Recall) and optimal thresholds for the top 20 most prevalent diseases in the 5-Year Prediction task (Task 4). Results correspond to the Large Model configuration using Data Split 1 and ICD-10 terminology.

Rank	N	Code	Disease Name	APS	Adj. Precision	Adj. Recall	Opt. Thresh
**1**	2228	I10	Essential (primary) hypertension	0.098	0.093	0.948	0.0905
**2**	1672	Z864	Personal history of psychoactive substance abuse	0.073	0.069	0.995	0.0696
**3**	1503	E780	Pure hypercholesterolaemia	0.065	0.062	0.999	0.0617
**4**	1331	M199	Arthrosis, unspecified	0.064	0.061	0.62	0.0548
**5**	1320	K573	Diverticular disease of large intestine without perforation or abscess	0.058	0.059	0.727	0.0562
**6**	1223	Z871	Personal history of diseases of the digestive system	0.053	0.051	0.962	0.05
**7**	1211	K449	Diaphragmatic hernia without obstruction or gangrene	0.046	0.05	0.998	0.0484
**8**	1192	Z922	Personal history of long-term (current) use of other medicaments	0.045	0.049	0.985	0.0491
**9**	1184	Z867	Personal history of diseases of the circulatory system	0.048	0.049	0.995	0.0477
**10**	1005	K219	Gastro-oesophageal reflux disease without oesophagitis	0.042	0.042	0.965	0.0412
**11**	919	R074	Chest pain, unspecified	0.037	0.038	0.999	0.0365
**12**	906	Z921	Personal history of long-term (current) use of anticoagulants	0.053	0.059	0.397	0.0371
**13**	891	M139	Arthritis, unspecified	0.033	0.037	0.99	0.0387
**14**	886	I259	Chronic ischaemic heart disease, unspecified	0.059	0.07	0.291	0.0376
**15**	879	Z966	Presence of orthopaedic joint implants	0.04	0.041	0.61	0.0351
**16**	852	K297	Gastritis, unspecified	0.036	0.036	0.803	0.0337
**17**	827	M179	Gonarthrosis, unspecified	0.028	0.034	0.995	0.0338
**18**	806	Z512	Other chemotherapy	0.038	0.041	0.622	0.0322
**19**	796	I209	Angina pectoris, unspecified	0.029	0.033	0.925	0.0318
**20**	792	Z538	Procedure not carried out for other reasons	0.041	0.049	0.241	0.0317

Supplementary Table S6. Illustrative example of a raw patient data, detailed by sequential hospital visits and associated clinical concepts (diagnoses and age). The codes are presented in CALIBER phenotype.

Patient		V1	V2	V3	V4	V5	V6	V7
1	Diagnosis	PH328, PH323, PH189	PH45	PH89, PH43	PH315, PH1027, PH329	PH167, PH152, PH158	PH182, PH1027	
	Age	65	67	72	74	75	82	
2	Diagnosis	PH117	PH121, PH204	PH1028				
	Age	45	67	72				
3	Diagnosis	PH53, PH152	PH292, PH93	PH154, PH158	[UNK]	PH77	PH85	PH215, PH329, PH57
	Age	53	55	58	62	63	65	74
4	Diagnosis	PH182, PH1027	PH230, PH39, PH189	PH182, PH179, PH189, PH323				
	Age	49	50	53				

Supplementary Table S7. Encoding representation of the illustrative patient sequence shown in Table S6, demonstrating the transformation of raw visits into model input.

Patient	Disease Encoding	Age Encoding
1	[[PH328,PH323,PH189],[PH45],[PH89,PH43],[PH315,PH107, PH329],[PH167,PH152,PH158],[PH182, PH1027]]	[[65,65,65],[67],[72,72],[74,74,74],[75,75,75],[82,82]]
2	[[PH117],[PH121, PH204],[PH1028]]	[[45],[67,67],[72]]
3	[[PH53,PH152],[PH292,PH93],[PH154,PH158],[UNK],[PH77] ,[PH85],[PH215, PH329, PH57]]	[[53,53],[55,55],[58,58],[62],[63],[65],[74,74,74]]
4	[[PH182, PH1027],[PH230, PH39, PH189],[PH182, PH179, PH189, PH323]]	[[49,49],[50,50,50],[53,53,53,53]]

Note on privacy: To ensure strict adherence to de-identification standards, examples on supplementary table S6 and S7 entirely synthetic. While the structural format mirrors our actual data processing, the specific combination of CALIBER codes and age trajectories was generated solely for illustrative purposes.
